# Age-Specific Ultrasound Reference Data for the Laterolateral Rectal Diameter in Healthy Children

**DOI:** 10.3390/diagnostics16193236

**Published:** 2026-10-07

**Authors:** Aleksandra Pucar, Sofija Cvejić, Darko Laketić, Dubravka Milutinović, Luka Đjoković, Teodora Pecarski, Matija Bećirović, Radmila Balaban Đurević, Zoran Radojičić, Nataša Milić

**Affiliations:** 1 Institute of Anatomy “Niko Miljanic”, Faculty of Medicine, University of Belgrade, 11000 Belgrade, Serbia; 2 Department of Radiology, University Children’s Hospital, 11000 Belgrade, Serbia; 3 Faculty of Medicine, University of Belgrade, 11000 Belgrade, Serbia; 4 National Heart and Lung Institute, Imperial College London, London SW3 6LY, UK; 5 Department of Anatomy, Faculty of Medicine Foca, University of East Sarajevo, 73300 Foča, Bosnia and Herzegovina; 6 Department of Urology, University Children’s Hospital, 11000 Belgrade, Serbia; 7 Institute for Medical Statistics and Informatics, Faculty of Medicine, University of Belgrade, 11000 Belgrade, Serbia

**Keywords:** laterolateral rectal diameter, pediatric ultrasound, healthy children, reference values, empirical reference data, transabdominal ultrasonography, functional constipation, percentile values, rectum

## Abstract

**Background:** Transabdominal ultrasonographic measurement of rectal diameter is increasingly used as a non-invasive adjunct for assessing rectal distension and fecal loading in children. However, interpretation is limited by the lack of age-stratified reference data in pediatric populations without bowel dysfunction. This study aimed to characterize the distribution of the laterolateral (LL) rectal diameter across childhood and adolescence and provide preliminary age-stratified empirical reference percentiles. **Methods:** This single-center cross-sectional study included pediatric participants aged ≤18 years who underwent clinically indicated abdominal ultrasonography for reasons unrelated to bowel or anorectal disorders and fulfilled predefined eligibility criteria. Maximal LL rectal diameter was measured transabdominally using a standardized protocol. Empirical percentiles were calculated for four age groups (6–36, 37–72, 73–144, and 145–216 months), with precision assessed using non-parametric bootstrap resampling with 10,000 repetitions. **Results:** The cohort comprised 188 participants: 46, 43, 62, and 37 in the four age groups, respectively; 107 (56.9%) were boys. Overall LL rectal diameter was 3.55 ± 1.46 cm (median, 3.20 cm; IQR, 2.50–4.30 cm), ranging from 1.01 to 8.00 cm. The LL diameter differed significantly across age groups (*p* = 0.002), with mean values increasing from 3.02 cm in the youngest group to 3.96 cm in the oldest group. No significant sex-related difference was observed (*p* = 0.843). P5–P95 ranges were 1.60–4.90, 1.51–5.89, 2.20–6.59, and 2.27–7.00 cm, respectively. Bootstrap confidence intervals were wider for extreme percentiles, particularly in smaller age strata. **Conclusions:** LL rectal diameter shows substantial interindividual variability and a modest age-related increase during childhood. These findings provide preliminary age-stratified empirical reference data rather than diagnostic thresholds or definitive normative limits. Larger multicenter studies are needed for external validation and more precise estimation of extreme percentiles.

## 1. Introduction

Functional constipation (FC) is a common gastrointestinal disorder in childhood and is frequently associated with fecal retention and rectal distension [1]. Chronic stool retention may lead to progressive rectal enlargement and altered rectal function, making the assessment of rectal dimensions potentially relevant in affected children. Rectal distension may also have implications beyond gastrointestinal symptoms, given the close relationship between bowel dysfunction and lower urinary tract symptoms within the spectrum of pediatric bladder–bowel dysfunction [2].

Transabdominal ultrasonography is a non-invasive, radiation-free, and readily repeatable method for assessing rectal distension in children. Measurement of the transverse or laterolateral (LL) rectal diameter has therefore been investigated as an objective marker of fecal retention and FC. Recent systematic reviews and meta-analyses have shown that rectal diameter is, on average, greater in children with FC than in unaffected controls [3,4,5]. However, considerable heterogeneity exists across studies, including differences in patient populations, ultrasonographic protocols, and proposed diagnostic thresholds. Reported cut-off values vary substantially, limiting the use of a single universal threshold in clinical practice [3,4].

A major limitation of the currently available evidence is the lack of standardized age-specific reference data from children without disorders expected to affect rectal morphology or bowel function. A recent systematic review identified the absence of age-dependent normal values, together with methodological heterogeneity, as an important barrier to the clinical interpretation of pediatric rectal ultrasonography [3]. The potential importance of age is further supported by prospective data from healthy infants demonstrating an increase in transverse rectal diameter during the first year of life [6]. These findings suggest that normal rectal dimensions may change throughout development and that age should be considered when interpreting rectal measurements in children.

Accordingly, the present study aimed to provide age-specific reference data and empirical percentile distributions for the LL rectal diameter measured by standardized transabdominal ultrasonography in a pediatric reference cohort without disorders expected to affect rectal morphology or bowel function. We additionally evaluated the association of LL rectal diameter with age and sex. By characterizing the physiological distribution of LL rectal diameter across childhood, this study was designed to provide a reference framework for pediatric rectal ultrasonography and to support future studies aimed at establishing clinically validated age-specific diagnostic thresholds.

## 2. Materials and Methods

### 2.1. Study Design and Setting

This single-center cross-sectional study was conducted at the University Children’s Hospital “Tiršova”, Belgrade, Serbia, between January 2024 and September 2026. The study combined prospectively obtained measurements with retrospective analysis of eligible measurements acquired during routine abdominal ultrasonography within the same study period. The study was conducted in accordance with the Declaration of Helsinki [7] and was approved by the Ethics Committee of the University Children’s Hospital, Belgrade, Serbia (Organizational Unit 017, Approval No. 16/137; 25 May 2026). The study was designed to characterize age-related reference distributions of the laterolateral (LL) rectal diameter in a pediatric reference population without disorders expected to affect rectal morphology or bowel function.

### 2.2. Study Population and Recruitment

Children and adolescents undergoing clinically indicated abdominal ultrasonography for reasons unrelated to bowel or anorectal disorders were assessed for eligibility. Thus, the study population represented a hospital-based pediatric reference cohort rather than a community-based sample. For prospectively assessed participants, eligibility criteria and examination conditions were documented using a standardized case report form (CRF). For the retrospective component, existing eligible ultrasonographic measurements and corresponding clinical records from the same study period were reviewed, and participants fulfilling the same eligibility criteria were included. No a priori sample-size calculation or predetermined recruitment target was used. All available participants fulfilling the eligibility criteria during the study period were considered for inclusion. Recruitment was not directed toward achieving equal or predefined numbers within individual age strata; consequently, the final sample size and the number of participants in each age category reflected the available eligible cohort.

### 2.3. Eligibility Criteria

Participants were eligible if they were 18 years of age or younger, had an adequate transabdominal ultrasonographic examination, and had no clinical condition expected to alter rectal morphology or bowel function. The standardized CRF used for prospective data collection specifically documented the absence of functional constipation, neurological disorders, anorectal anomalies, and previous surgery in the anorectal region. Functional constipation was defined according to the Rome IV criteria [8,9]. Additional exclusion criteria were congenital anorectal malformations, Hirschsprung disease, neurological disorders associated with bowel dysfunction, including spina bifida and cerebral palsy, endocrine or metabolic disorders known to affect gastrointestinal motility, a history of abdominal or pelvic surgery with potential effects on rectal anatomy or function, current laxative treatment, systemic connective tissue disease, and inadequate ultrasonographic visualization of the rectum. For retrospectively identified participants, eligibility was determined from the available medical records, recorded diagnoses, and ultrasonographic documentation according to the same study criteria.

### 2.4. Age Stratification

For the primary age-stratified analyses, four age categories were defined a priori: 6–36 months, 37–72 months, 73–144 months, and ≥145 months. These categories were defined according to age and independently of the subsequently observed number of participants or LL rectal diameter values within each group. Recruitment was not targeted to achieve predefined sample sizes within individual age strata. In addition to the four a priori age categories, a post hoc exploratory analysis was performed by combining participants into two broader age groups: 6–72 months and ≥73 months. This analysis was undertaken during statistical analysis to explore differences between broader age categories and was not intended to establish or validate a clinical age threshold.

### 2.5. Ultrasonographic Examination

All ultrasonographic examinations and LL rectal diameter measurements were performed by experienced pediatric radiologists using a standardized examination protocol. The radiologist was not blinded to participants’ age or to the general clinical indication for ultrasonography. The intraobserver and interobserver reliability for the maximal LL rectal diameter was excellent, with an ICC > 0.90. Transabdominal ultrasonography was performed using a Siemens Acuson S2000 ultrasound system (Siemens Medical Solutions USA Inc., Malvern, PA, USA) equipped with a 2–6 MHz convex transducer. Participants were examined in the supine position. The transducer was positioned approximately 2 cm above the pubic symphysis, and the urinary bladder was adequately filled to provide an acoustic window for visualization of the rectum. The examination technique was based on the previously described transabdominal approach for pediatric rectal diameter measurement [10] and is consistent with the methodology summarized in a recent systematic review [3]. For prospectively assessed participants, patient position and adequacy of bladder filling were documented on the standardized CRF.

### 2.6. Measurement Protocol

**Figure 1:**
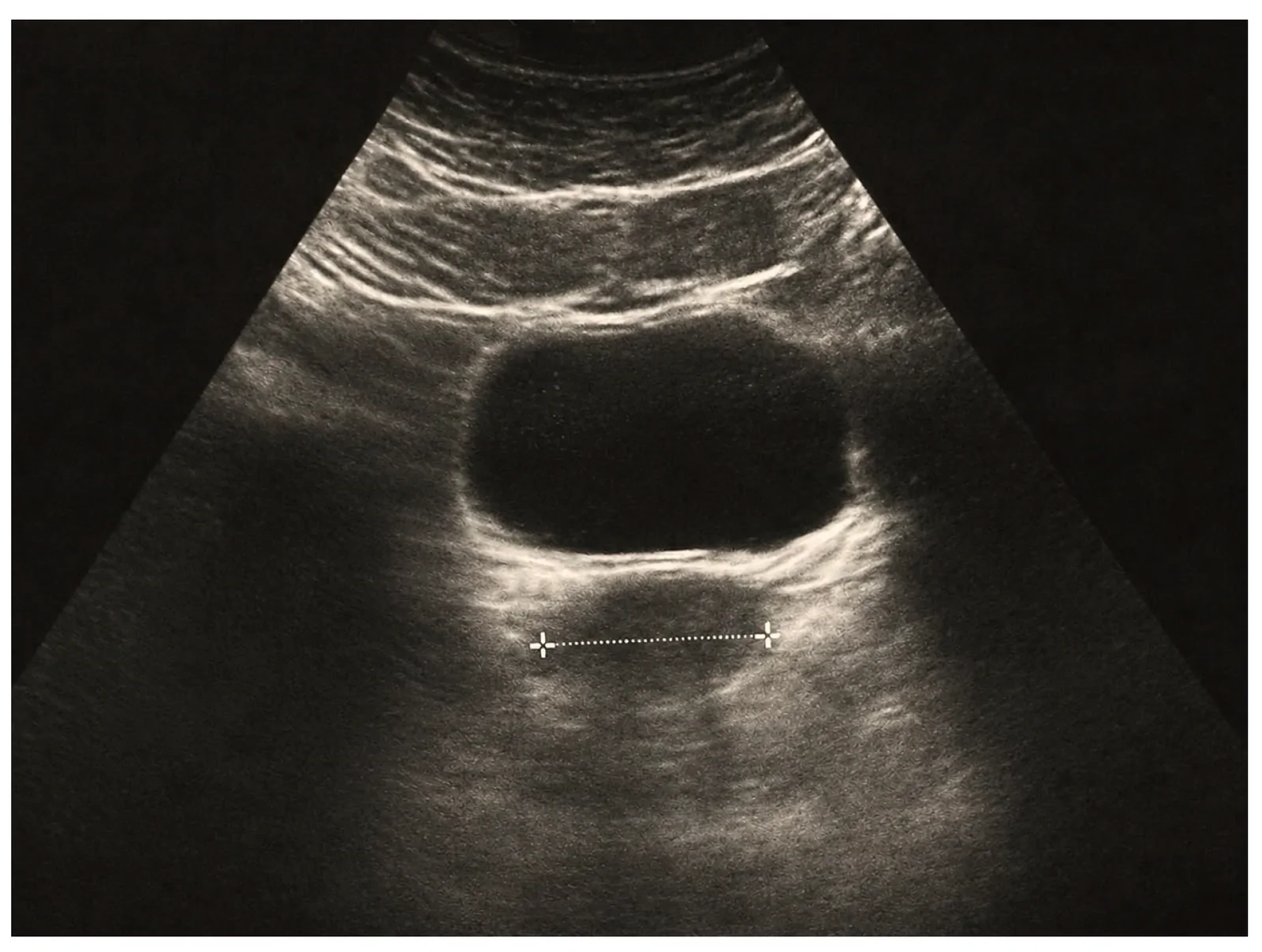
Ultrasonographic measurement of the lateral-to-lateral (LL) diameter of the rectum.

The transducer was angled approximately 15° caudally from the transverse plane to visualize the rectum posterior to the urinary bladder. The LL rectal diameter was defined as the maximum distance between the outer contours of the rectal wall in the axial plane [10] (Figure 1). Calipers were positioned on the external borders of the rectal wall, perpendicular to the maximal LL diameter. The time elapsed since the last bowel movement and the presence of an urge to defecate at the time of examination were documented because transabdominal rectal diameter measurements may vary in relation to defecation [11]. According to the predefined study protocol, measurement was postponed if defecation had occurred within the preceding 2 h or if the participant reported an urgent need to defecate.

### 2.7. Data Collection and Anonymization

For each participant, age at examination, sex, and LL rectal diameter were recorded. For prospectively assessed participants, eligibility criteria, relevant examination conditions, and LL rectal diameter measurements were documented using the standardized CRF. For retrospectively identified participants, eligible measurements and corresponding demographic and clinical information were extracted from existing institutional records. All data were anonymized before statistical analysis, and no directly identifying information was included in the analytical dataset.

### 2.8. Statistical Analysis

Statistical analyses were performed using IBM SPSS Statistics, version 29.0 (IBM Corp., Armonk, NY, USA). Continuous variables are presented as mean ± standard deviation (SD) and median with interquartile range (IQR), as appropriate, whereas categorical variables are presented as absolute frequencies and percentages. Differences among the four age groups were evaluated using the one-way ANOVA with the Least Significant Difference (LSD) test for post hoc comparisons. The magnitude of the between-group effect was quantified using epsilon-squared (ε^2^). Comparisons between male and female participants were performed using Student’s *t*-test. The comparison between the two broader age categories was considered a post hoc exploratory analysis and was also performed using Student’s *t*-test. The magnitude of the between-group effect was quantified using Cohen’s d (d). The correlation between age and LL rectal diameter was evaluated using Pearson’s correlation coefficient. Simple linear regression was used to estimate the mean change in LL rectal diameter per additional month of age. A multivariable linear regression model including age and sex was then fitted to evaluate whether age remained independently associated with LL rectal diameter after adjustment for sex. Regression coefficients are reported with 95% confidence intervals (CIs), *p*-values, and coefficients of determination (R^2^). Reference values were derived empirically because the outcome distribution deviated from normality. The 5th, 10th, 25th, 50th, 75th, 90th, and 95th percentiles (P5, P10, P25, P50, P75, P90, and P95) were calculated for each of the four a priori age groups. To quantify the precision of percentile estimates, 95% confidence intervals were obtained using non-parametric bootstrap resampling with 10,000 repetitions performed separately within each a priori age stratum. Given the limited number of participants within individual age strata, particularly for the estimation of distribution tails, extreme percentiles (P5 and P95) were interpreted cautiously and were not considered diagnostic thresholds. Percentile values for the two broader post hoc age categories were additionally calculated for exploratory purposes. The dataset was screened for missing data before statistical analysis, and only complete cases were included. All statistical tests were two-sided, and *p* < 0.05 was considered statistically significant.

### 2.9. Sample Size

Given the observed sample size of 188 participants across four groups (*n* = 46, 43, 62, and 37), a sensitivity power analysis for a one-way ANOVA indicated that the study had 80% power to detect a small to medium effect size (Cohen’s f = 0.24) at a significance level of α = 0.05.

## 3. Results

### 3.1. Characteristics of the Study Population and Overall LL Rectal Diameter

The pediatric cohort comprised 188 participants aged 6–212 months. There were 107 boys (56.9%) and 81 girls (43.1%). Median age was 79.5 months (IQR, 37.75–136.00 months). The overall LL rectal diameter was 3.55 ± 1.46 cm, with a median of 3.20 cm (IQR, 2.50–4.30 cm) and a range of 1.01–8.00 cm.

### 3.2. LL Rectal Diameter Across the Four a Priori Age Groups

**Figure 2:**
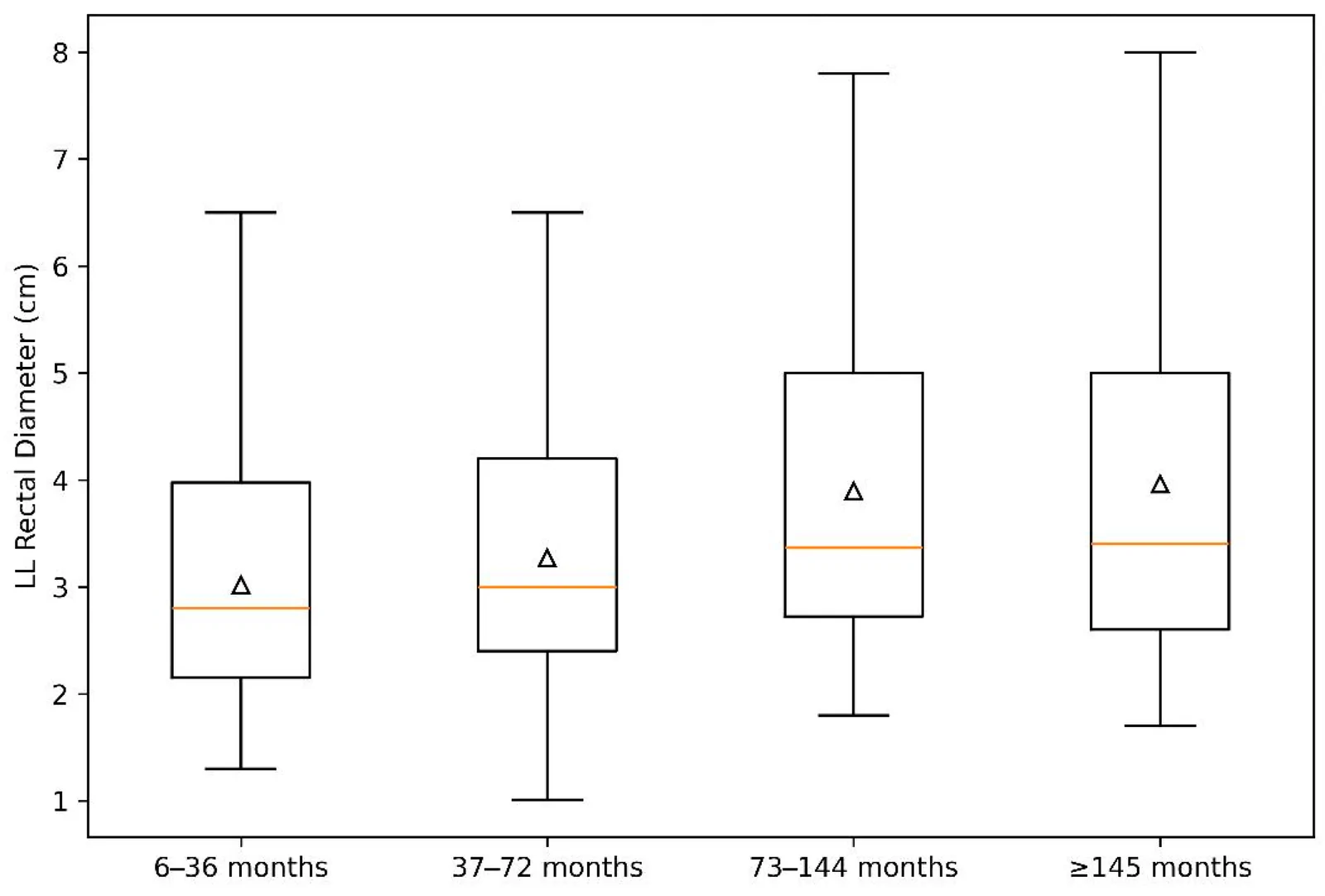
Box plot of the LL rectal diameter across the four a priori age groups. The horizontal line within each box represents the median, the box indicates the interquartile range, and the triangle represents the mean value.

Participants were categorized into four age groups according to age in months: 6–36 months (group 1: *n* = 46), 37–72 months (group 2: *n* = 43), 73–144 months (group 3: *n* = 62), and group 4: 145–216 months (*n* = 37). The analysis according to age groups demonstrated a gradual increase in LL rectal diameter with increasing age. Mean LL rectal diameter increased from 3.02 cm in the youngest group to 3.96 cm in the oldest group (Table 1 and Figure 2). The LL diameter differed significantly across the four age groups (*p* = 0.002). Post hoc pairwise comparisons showed significant differences between groups 1 and 3 (*p* = 0.001), groups 1 and 4 (*p* = 0.003), groups 2 and 3 (*p* = 0.031), and groups 2 and 4 (*p* = 0.041), indicating that the differences were primarily between the younger (groups 1 and 2) and older age groups (groups 3 and 4). The corresponding effect size was small (ε^2^ = 0.061).

### 3.3. Post Hoc Exploratory Comparison of Two Broader Age Groups

**Figure 3:**
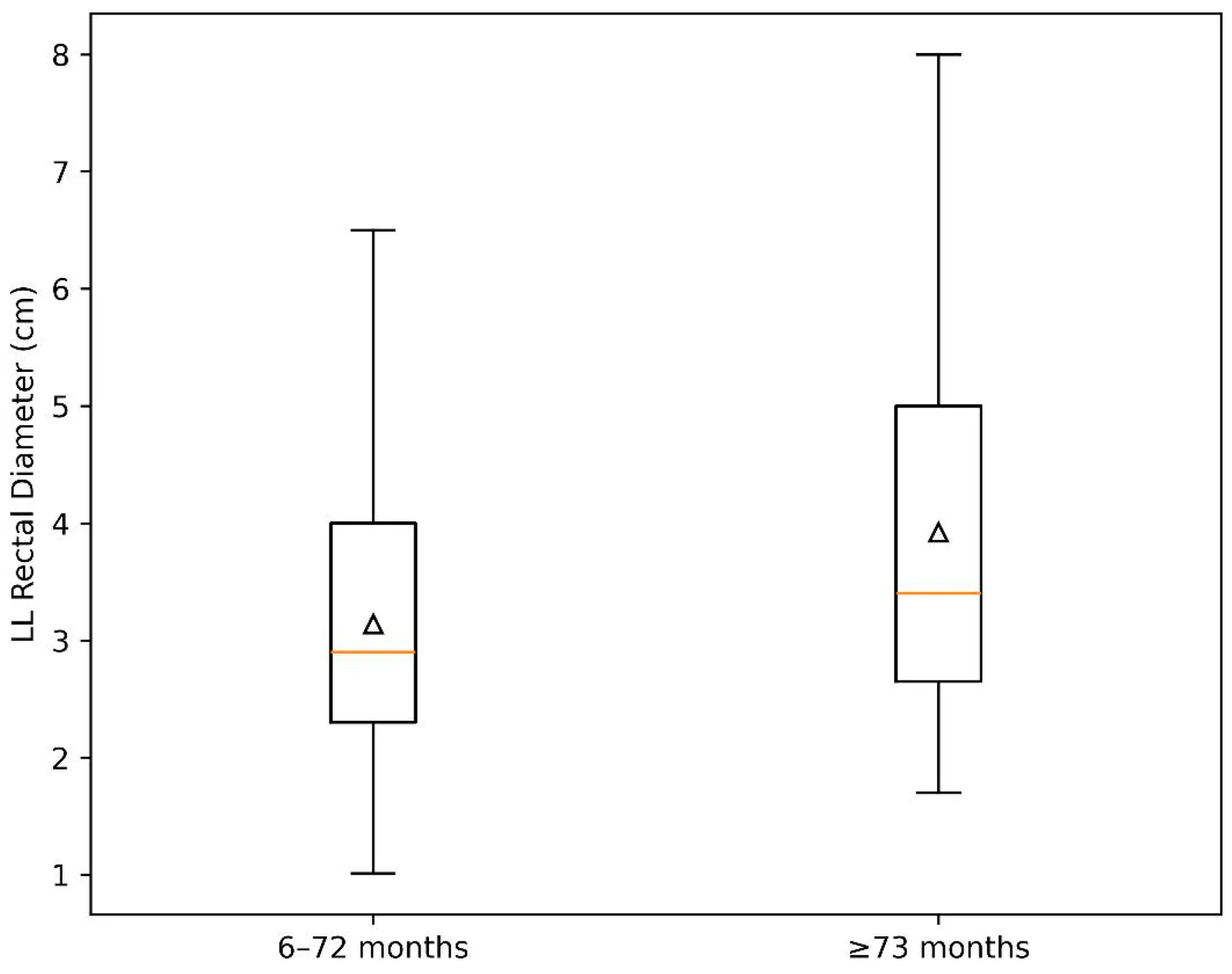
Box plot of the LL rectal diameter of two broader age groups. The horizontal line within each box represents the median, the box indicates the interquartile range, and the triangle represents the mean value.

In the post hoc exploratory analysis, participants aged ≥73 months had larger LL rectal diameters than those aged 6–72 months (*p* < 0.001) (Table 2 and Figure 3). Because this age dichotomization was defined during the analysis rather than a priori, this finding should be interpreted as exploratory and does not represent a validated clinical age threshold. The Cohen’s *d* effect size was 0.555, indicating a moderate effect.

### 3.4. LL Rectal Diameter According to Sex

**Figure 4:**
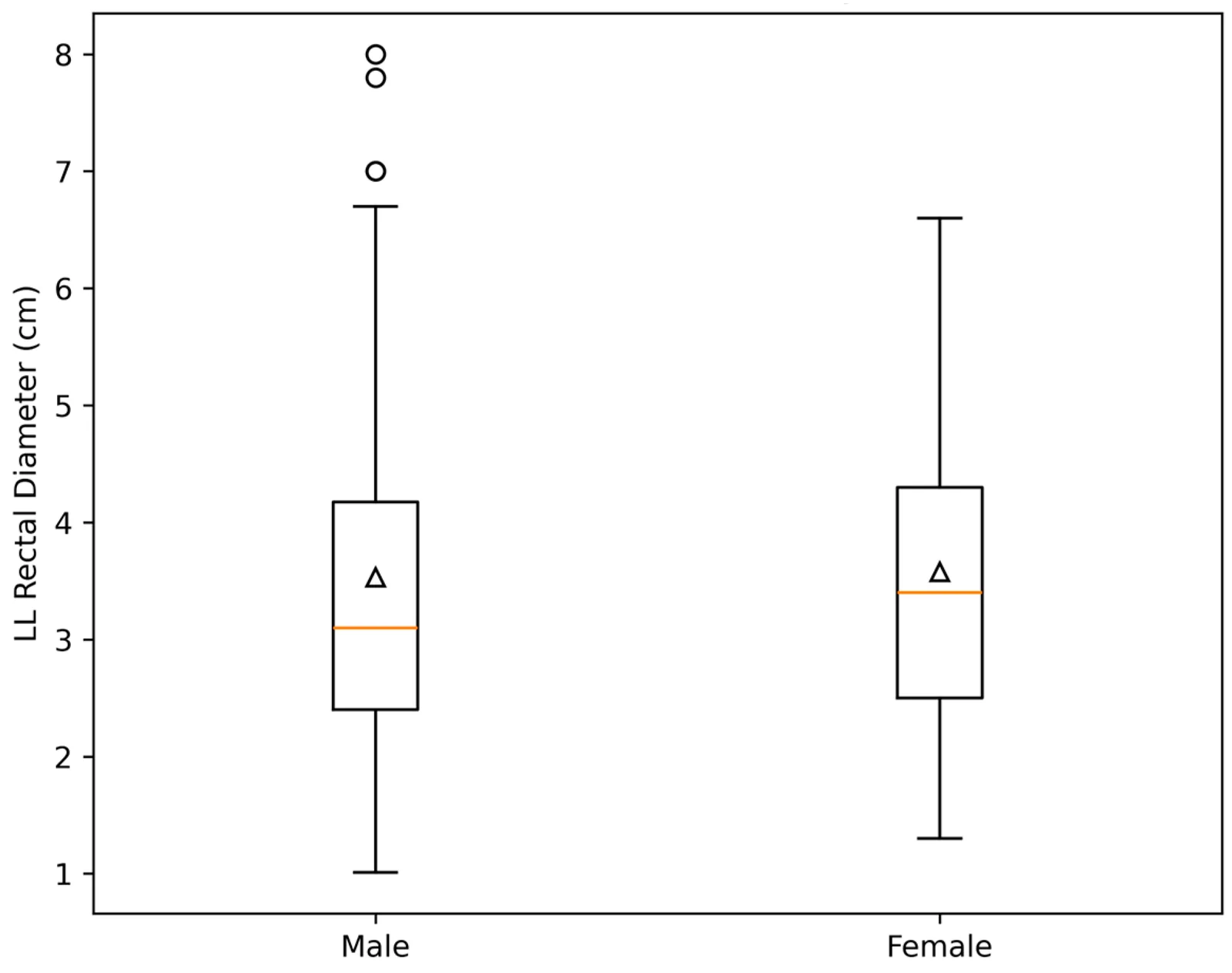
Box plot of the LL rectal diameter according to sex. The horizontal line within each box represents the median, the box indicates the interquartile range, and the triangle represents the mean value. Circle represents outliers.

No statistically significant sex-related difference in LL rectal diameter was observed between boys and girls (*p* = 0.843). The mean LL rectal diameter was 3.53 cm in boys and 3.58 cm in girls (Table 3 and Figure 4). The corresponding Cohen’s d effect size was very small (d = 0.029). When stratified by the four a priori-defined age groups, no statistically significant differences in LL rectal diameter were observed between boys and girls in any age group (all *p* > 0.05). Detailed results of LL rectal diameter according to sex are presented in Supplementary Table S1.

### 3.5. Association Between LL Rectal Diameter and Age

**Figure 5:**
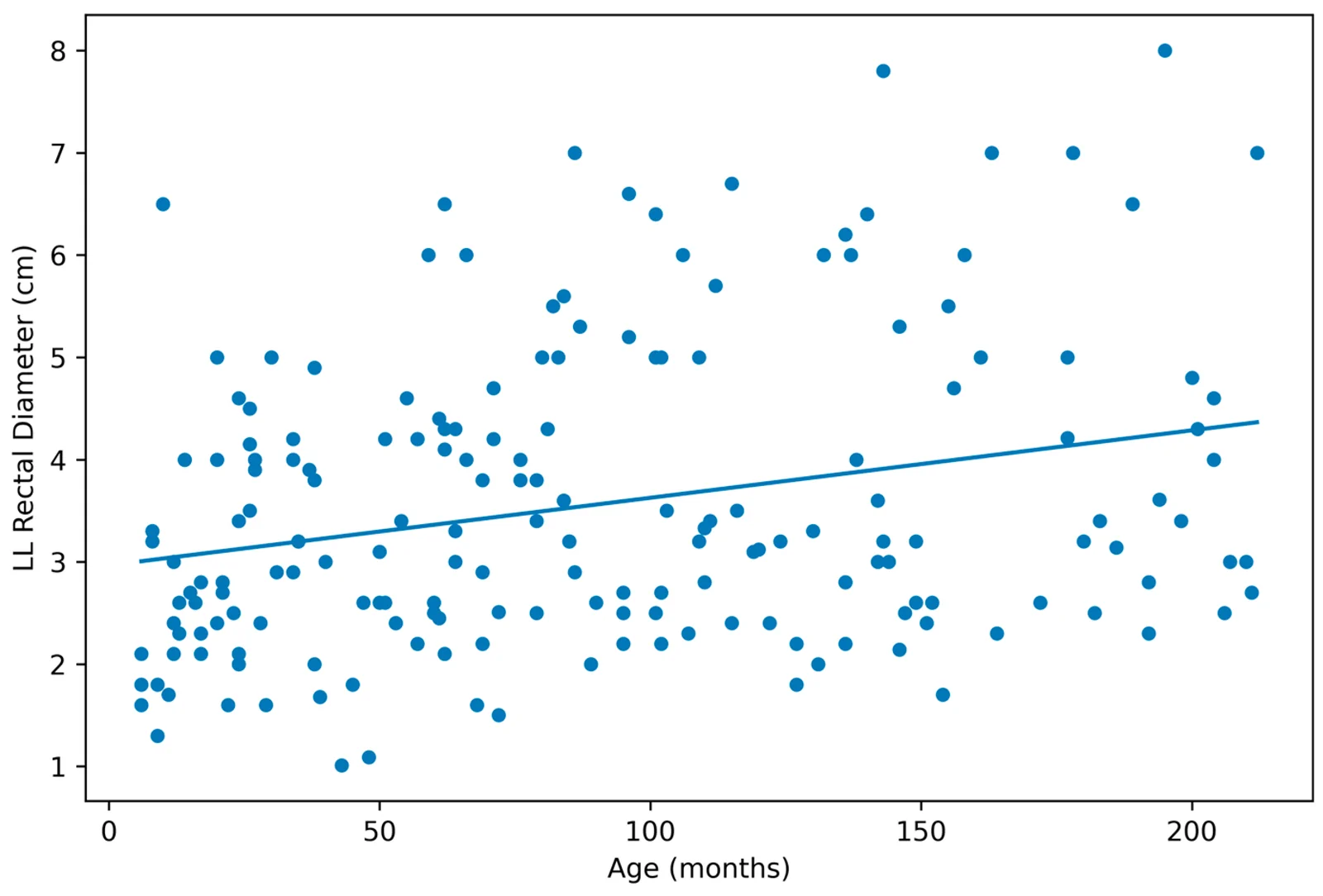
Scatter plot illustrating the relationship between age and LL rectal diameter with the fitted linear regression line.

Age showed a weak positive correlation with LL rectal diameter (r = 0.264, *p* < 0.001). In simple linear regression, LL rectal diameter increased by 0.0066 cm per month of age (95% CI, 0.003–0.010; *p* < 0.001) (Table 4 and Figure 5). In the multivariable model including age and sex, age remained independently associated with LL rectal diameter, whereas sex was not significant. The model explained approximately 7% of the variance in LL rectal diameter (R^2^ = 0.070), indicating that LL rectal diameter increases with age, although substantial inter-individual variability exists among children of the same age.

### 3.6. Age-Specific Empirical Percentiles of the LL Rectal Diameter

**Figure 6:**
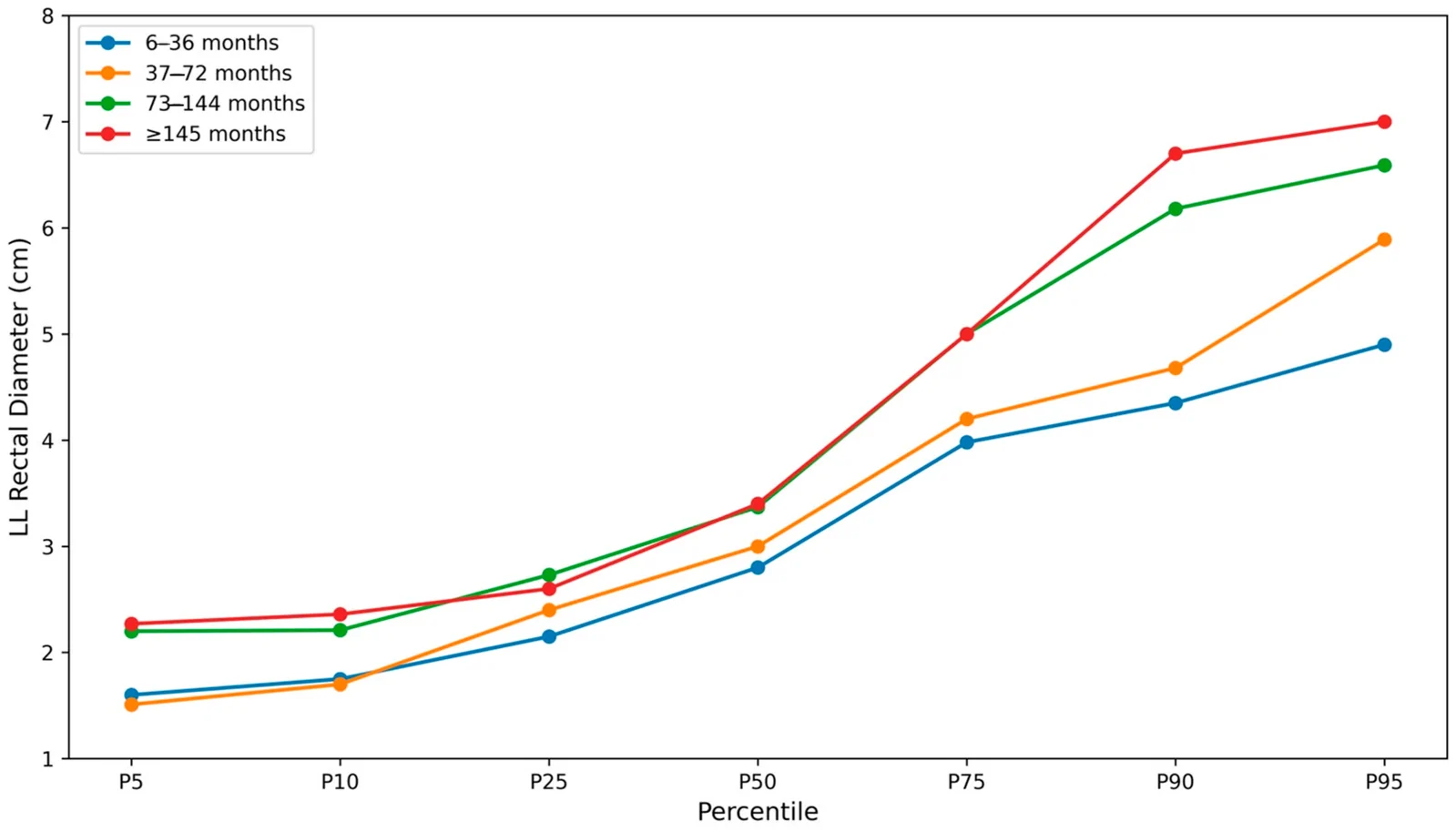
Age-specific empirical percentiles of the LL rectal diameter.

To facilitate clinical interpretation, empirical percentile values of the LL rectal diameter were calculated for each age group. The median (P50) was 2.80 cm in children aged 6–36 months, 3.00 cm in those aged 37–72 months, 3.37 cm in the 73–144-month group, and 3.40 cm in children aged ≥145 months. The upper percentile values also increased with age, with the 95th percentile reaching 4.90 cm, 5.89 cm, 6.59 cm, and 7.00 cm across the four age groups, respectively (Table 5 and Figure 6). These findings indicate that a single universal cut-off value for all pediatric age groups may be overly simplistic and potentially inaccurate, particularly when comparing young children with adolescents.

Empirical percentile estimates increased generally with age, particularly in the upper part of the distribution. To quantify the precision of these estimates, non-parametric bootstrap 95% confidence intervals were calculated using 10,000 resamples within each a priori age stratum (Table 6).

Bootstrap confidence intervals were wider for the extreme percentiles, particularly P95 in the smaller age strata. These findings indicate greater uncertainty in the tails of the age-specific distributions; therefore, P5 and P95 are presented as preliminary empirical age-specific reference estimates rather than fixed normative limits or diagnostic thresholds.

**Figure 7:**
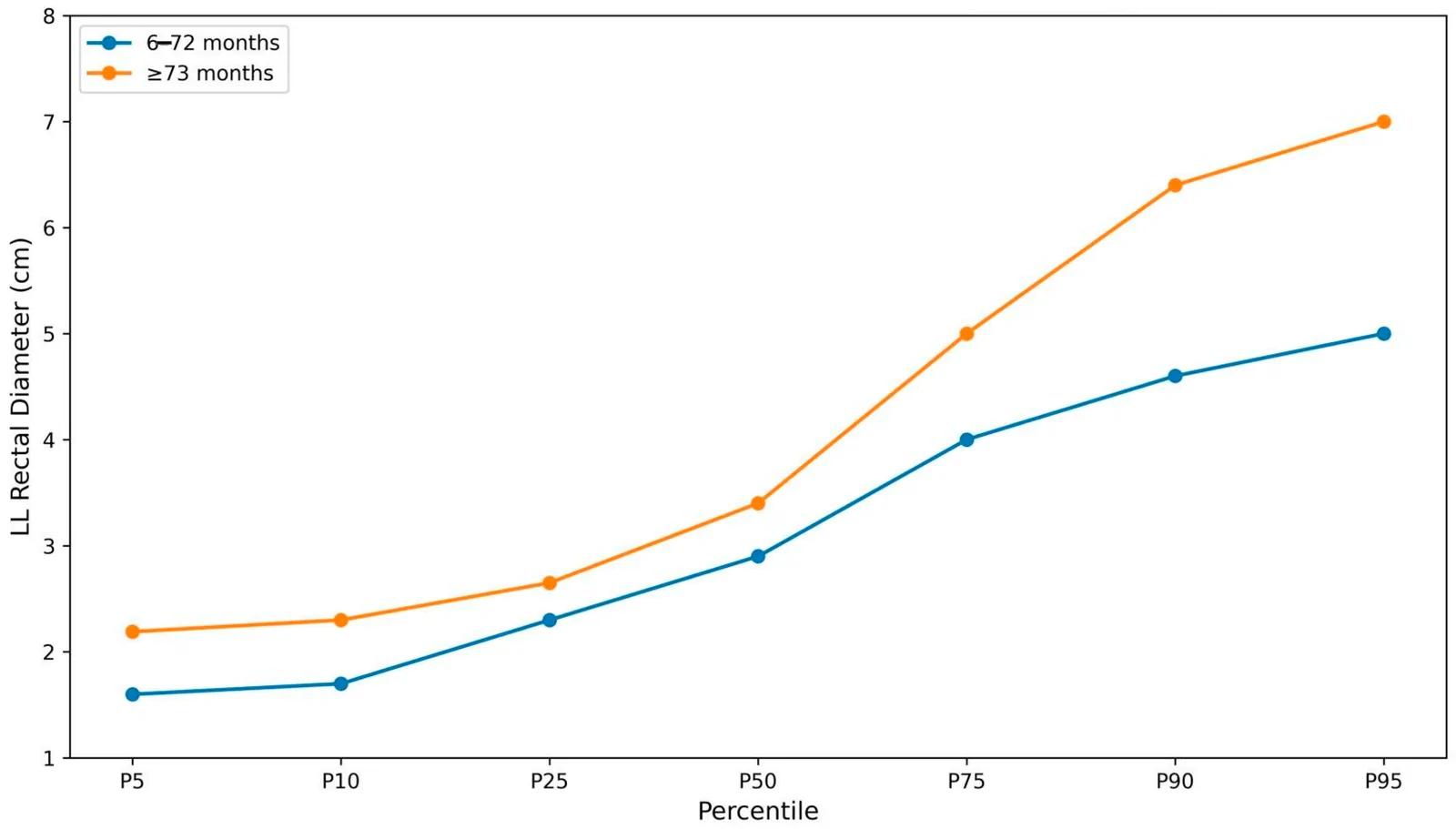
Percentile values of the LL rectal diameter according to two age groups.

For simplified clinical application, empirical percentile values were additionally calculated for two age groups (6–72 months and ≥73 months). The median (P50) was 2.90 cm in children aged 6–72 months and 3.40 cm in those aged ≥73 months. Higher percentile values were consistently observed in the older age group, with the 95th percentile increasing from 5.00 cm to 7.00 cm (Table 7 and Figure 7). Percentiles for the two broader age categories are provided for exploratory description only and should not be interpreted as evidence supporting a clinical cut-off at 73 months.

## 4. Discussion

### 4.1. Principal Findings

This study provides age-stratified empirical reference data for the laterolateral (LL) rectal diameter measured by transabdominal ultrasonography in a hospital-based pediatric cohort without disorders expected to affect rectal morphology or bowel function. Four principal findings emerged. First, LL rectal diameter showed a non-normal distribution and substantial interindividual variability. Second, age was positively associated with LL rectal diameter, and the four a priori age groups differed significantly overall. Third, no significant sex-related differences in LL rectal diameter were identified. Finally, the upper percentiles of the empirical distribution tended to increase with age, supporting age-aware interpretation rather than reliance on a single universal value. Because confidence intervals were wider for the extreme percentiles, particularly in the smaller age strata, P5 and P95 should be regarded as preliminary empirical reference estimates rather than fixed limits of normality.

The positive association between age and LL rectal diameter observed in the present study is biologically plausible and consistent with previous pediatric literature. Progressive growth of the pelvis, maturation of anorectal function, and increasing rectal reservoir capacity are expected to influence rectal dimensions throughout childhood. Gatzinsky et al. reported developmental variation in rectal diameter during infancy, emphasizing the need for age-specific interpretation in young children [6]. Our findings extend this concept across later childhood and adolescence by providing empirical reference data over a broader age range.

Previous ultrasonographic studies primarily investigated functional constipation, fecal retention, and fecal impaction, frequently reporting larger rectal diameters in affected children than in comparison groups [10,12,13,14,15,16,17,18]. Although these studies established the clinical relevance of rectal ultrasonography, they did not provide comprehensive age-stratified normative reference values. Recent systematic reviews likewise concluded that methodological heterogeneity and the lack of standardized age-specific reference data remain major barriers to wider clinical implementation [3,4].

Although age was an independent predictor of LL rectal diameter, the relatively low coefficient of determination indicates that age alone explains only a small proportion of the observed variability. Rectal dimensions may be influenced by stool burden, bowel habits, timing relative to defecation, and technical aspects of the measurement protocol [3,4,11]. Individual anatomical variation may contribute to the remaining unexplained variability. Additional factors such as body size, hydration, dietary intake, rectal compliance, and pelvic floor function may also contribute, although these were not evaluated in the present study. In the present cohort, substantial overlap was observed between adjacent age groups. This supports the use of age-specific reference distributions rather than a single universal threshold, consistent with concerns raised in recent systematic reviews [3,4].

### 4.2. Sex-Related Differences

No statistically significant sex-related differences in LL rectal diameter were observed in the overall cohort, either in the univariable analysis or after adjustment for age in the multivariable model. Furthermore, when boys and girls were compared separately within each of the four age groups, no significant differences in LL rectal diameter were identified in any age stratum. These findings indicate that the absence of an overall sex effect was not merely attributable to differences in age distribution between boys and girls. The substantial overlap between male and female distributions suggests that sex contributes little to the variability of LL rectal diameter compared with age. Accordingly, the present findings do not support the need for separate sex-specific reference percentiles in this cohort. Previous pediatric ultrasonographic studies have focused primarily on functional constipation, fecal retention, and treatment response, whereas sex-specific normative differences have received minimal attention [12,16,17,18]. Similarly, recent systematic reviews primarily identified methodological and clinical heterogeneity as major sources of between-study variability [3,4]. Our multivariable analysis further supports this observation by demonstrating that sex does not independently influence LL rectal diameter after accounting for age. These findings should be interpreted in light of several considerations. The number of participants in late adolescence was relatively small, pubertal status was not available, and anthropometric measurements were not included in the analysis. Future multicenter studies incorporating pubertal development and body-size parameters are warranted to determine whether subtle sex-related differences emerge after maturation.

### 4.3. Clinical Relevance of Age-Specific Percentile Values

The empirical percentile distributions generated in the present study represent an important contribution to clinical practice. Unlike fixed diagnostic thresholds, percentile-based reference values allow for age-related variation in rectal size and interpretation of an individual measurement within the expected distribution for a given age. In the present cohort, the empirical 95th percentile showed a general age-related increase from the youngest to the oldest age group, although uncertainty around the extreme percentile estimates was substantial. An age-related increase in rectal diameter has also been reported during the first year of life [6].

These findings help explain the variability in previously proposed diagnostic cut-off values. Earlier studies evaluated heterogeneous patient populations, used different ultrasonographic protocols and anatomical landmarks, and primarily aimed to distinguish children with functional constipation from healthy controls [10,12,13,14,15,16,17,18]. Consequently, suggested cut-off values varied substantially between studies [3,4]. Recent systematic reviews similarly concluded that the absence of standardized measurement protocols and age-specific normative data limits the clinical applicability of rectal ultrasonography [3,4].

Age-specific percentiles and diagnostic cut-offs should therefore be regarded as complementary rather than interchangeable concepts. Percentiles describe the physiological distribution of LL rectal diameter in children in a reference cohort, whereas diagnostic thresholds are intended to discriminate between health and disease. The empirical reference values presented in this study provide a physiological framework that may improve interpretation of pediatric rectal ultrasonography and support future studies aimed at defining clinically validated diagnostic thresholds. These findings address the need for standardized normative data highlighted in recent systematic reviews [3,4].

The bootstrap analysis performed in our study provides an estimate of the uncertainty surrounding the empirical percentiles. Confidence intervals were wider at the distribution tails, particularly for P95 in the smaller age strata. This is expected because extreme percentiles are sensitive to sample size and individual extreme observations. Accordingly, P5 and P95 should be interpreted cautiously. The more central percentiles provide a more stable description of the observed distribution, whereas the extreme percentiles should primarily serve as preliminary reference estimates for future validation rather than as clinical decision limits.

Nevertheless, the proposed percentile values should currently be considered preliminary empirical reference data rather than diagnostic criteria or definitive normative limits. Because symptomatic children were not included and diagnostic accuracy was not evaluated, external validation in multicenter prospective studies is required before specific percentile thresholds can be incorporated into routine clinical decision-making.

### 4.4. Clinical Implications for Functional Constipation, Bladder–Bowel Dysfunction, and Neurourology

Functional constipation remains a clinical diagnosis based on clinical history, physical examination, and the Rome IV criteria [8,9]. Although the 2014 ESPGHAN/NASPGHAN guideline did not recommend routine rectal ultrasonography because of insufficient supporting evidence [19], accumulating data from subsequent studies and recent meta-analyses have consistently demonstrated that children with functional constipation and fecal impaction tend to have larger rectal diameters than healthy controls [3,4,16,17,18].

In this context, transabdominal ultrasonography represents a potentially valuable adjunct rather than a replacement for clinical assessment. Its non-invasive nature and absence of ionizing radiation may be particularly advantageous when digital rectal examination is declined, undesirable, or provides inconclusive findings [3,4,15,19].

The age-specific reference percentiles provided in the present study may improve the interpretation of rectal ultrasonography by allowing individual measurements to be evaluated within the observed distribution for a given age. Rather than relying on a single universal cut-off value, clinicians may identify children whose rectal diameter exceeds the expected distribution and who therefore warrant further clinical evaluation. Such an approach could potentially reduce age-related misclassification; however, this hypothesis requires validation in symptomatic cohorts.

Beyond functional constipation, these empirical reference data may also facilitate future research into bladder–bowel dysfunction and pediatric neurourology [20,21,22,23,24], as well as colorectal disorders in which ultrasonographic assessment of fecal load may be clinically relevant [25]. However, the present percentile values should be regarded as physiological preliminary empirical reference data rather than validated diagnostic thresholds. Prospective multicenter studies including symptomatic populations are required before these reference values can be incorporated into routine diagnostic algorithms.

### 4.5. Strengths and Limitations

To our knowledge, this study is among the first to provide age-stratified empirical reference data and percentile distributions for the LL rectal diameter across a broad pediatric age range using a standardized transabdominal ultrasonographic protocol. The main strengths of this study are the broad pediatric age range, standardized transabdominal measurement protocol, predefined age strata, and exclusion of disorders expected to alter rectal morphology or bowel function. The study also reports the full empirical distribution rather than only means or a single proposed threshold. Bootstrap confidence intervals were additionally calculated to quantify the precision of the percentile estimates and to address uncertainty in the distribution tails.

Several limitations should be acknowledged. First, this was a single-center, hospital-based reference cohort rather than a population-based sample, which may limit the generalizability of the findings. Second, the cross-sectional design describes between-participant age-related differences but cannot characterize individual developmental trajectories. Third, although the cohort covered a wide pediatric age range, the number of participants within individual strata was limited, particularly in the oldest age group. The smaller subgroup sizes reduced the precision of extreme percentile estimates, as reflected by the wider bootstrap confidence intervals. Although no sex-related differences were detected within individual age strata, these subgroup analyses involved smaller numbers of participants and may have limited power to detect subtle sex-related effects. Fourth, anthropometric variables, pubertal status, dietary habits, stool consistency, and bowel frequency were not analyzed and may account for part of the unexplained variability in LL rectal diameter. Finally, the study did not include children with functional constipation or other symptomatic bowel disorders and included only asymptomatic participants; therefore, diagnostic performance and optimal clinical cut-off values could not be determined. Despite these limitations, the present study provides preliminary age-stratified empirical reference data that address an important gap identified in recent systematic reviews [3,4]. External validation in larger multicenter cohorts and prospective studies including symptomatic children will be essential to confirm the generalizability and clinical applicability of these reference values.

### 4.6. Future Directions

Future work should prioritize multicenter validation using a harmonized ultrasound protocol. Standardization should specify patient position, bladder filling, probe type and frequency, anatomical measurement level, caliper placement, timing relative to defecation, and the number of repeated measurements. Reporting both absolute diameters and age-specific percentile positions would improve comparability across studies. Larger cohorts should support continuous reference curves rather than broad age categories. Methods used for pediatric growth references, including generalized additive models for location, scale, and shape, could generate smoothed centile curves across age. Incorporating height, weight, body mass index, body surface area, and pubertal stage may improve prediction and determine whether age or body size is the more appropriate scaling variable. Prospective diagnostic studies should then compare age-adjusted percentiles with Rome IV diagnoses, digital rectal examination, stool burden, symptom severity, and treatment response. Separate validation is required in functional constipation, bladder–bowel dysfunction, anorectal malformations, Hirschsprung disease, and neurogenic bowel populations. Longitudinal studies should evaluate within-child change, measurement reliability, and whether movement across percentile bands predicts clinical improvement or relapse. Ultimately, externally validated age-adjusted percentile curves may help define the potential role of rectal ultrasonography in pediatric clinical assessment.

## 5. Conclusions

This study provides age-stratified empirical reference data and percentile distributions for the LL rectal diameter measured by transabdominal ultrasonography in a pediatric reference cohort. LL rectal diameter tended to increase with age, whereas no independent association with sex was identified. These findings support age-aware interpretation of LL rectal diameter rather than reliance on a single universal threshold when interpreting pediatric rectal ultrasonography. The proposed empirical percentile values provide a physiological reference framework for the interpretation of LL rectal diameter and may serve as a basis for future research on functional constipation, bladder–bowel dysfunction, and pediatric neurourology. External validation in larger multicenter cohorts, including symptomatic populations, is required before the relationship between these reference distributions and clinically useful diagnostic thresholds can be established.

## Figures and Tables

**Table 1:** LL rectal diameter across the four a priori age groups.

Age Group	*n*	Mean ± SD	Median (IQR)	Range
6–36 months	46	3.02 ± 1.10	2.80 (2.15–3.98)	1.30–6.50
37–72 months	43	3.27 ± 1.31	3.00 (2.40–4.20)	1.01–6.50
73–144 months	62	3.90 ± 1.53	3.37 (2.73–5.00)	1.80–7.80
≥145 months	37	3.96 ± 1.65	3.40 (2.60–5.00)	1.70–8.00

**Table 2:** LL rectal diameter of two broader age groups.

Age Group	*n*	Mean ± SD	Median (IQR)	Range
6–72 months	89	3.14 ± 1.21	2.90 (2.30–4.00)	1.01–6.50
≥73 months	99	3.92 ± 1.57	3.40 (2.65–5.00)	1.70–8.00

**Table 3:** LL rectal diameter according to sex.

Sex	*n*	Mean ± SD	Median (IQR)	Range
Male	107	3.53 ± 1.59	3.10 (2.40–4.18)	1.01–8.00
Female	81	3.58 ± 1.27	3.40 (2.50–4.30)	1.30–6.60

**Table 4:** Regression analysis for age, sex, and LL rectal diameter.

Analysis	B	95% CI	*p* Value
Age, months	0.007	0.003–0.010	0.001
Male Sex	−0.029	−0.440–0.382	0.889

**Table 5:** Age-specific empirical percentiles of the LL rectal diameter.

Age Group	P5	P10	P25	P50	P75	P90	P95
6–36 months	1.60	1.75	2.15	2.80	3.98	4.35	4.90
37–72 months	1.51	1.70	2.40	3.00	4.20	4.68	5.89
73–144 months	2.20	2.21	2.73	3.37	5.00	6.18	6.59
≥145 months	2.27	2.36	2.60	3.40	5.00	6.70	7.00

**Table 6:** Age-specific non-parametric bootstrap 95% confidence intervals for P5 and P95 of the LL rectal diameter based on 10,000 resamples within each a priori age stratum.

Age Group	P5 (95% CI), cm	P95 (95% CI), cm
6–36 months	1.60 (1.38–1.88)	4.90 (4.11–6.13)
37–72 months	1.51 (1.02–2.02)	5.89 (4.39–6.45)
73–144 months	2.20 (2.00–2.40)	6.59 (6.00–7.00)
145–216 months	2.27 (1.70–2.50)	7.00 (5.60–8.00)

**Table 7:** Percentile values of the LL rectal diameter according to two age groups.

Age Group	P5	P10	P25	P50	P75	P90	P95
6–72 months	1.60	1.70	2.30	2.90	4.00	4.60	5.00
≥73 months	2.19	2.30	2.65	3.40	5.00	6.40	7.00

## Data Availability

The data that support the findings of this study are available from the corresponding author upon reasonable request. The data are not publicly available because they contain clinical information subject to ethical and privacy restrictions.

## Supplementary Materials

The following supporting information can be downloaded at https://www.mdpi.com/article/10.3390/diagnostics16193236/s1, Table S1. Sex differences within each a priori age group.
