# Supplementary material for: Age-Specific Ultrasound Reference Data for the Laterolateral Rectal Diameter in Healthy Children
Source: Diagnostics (Basel). 2026 Oct 7;16(19):3236. doi: 10.3390/diagnostics16193236 (PMC13664373; doi:10.3390/diagnostics16193236)
Supplement: Supplementary file 1 [file diagnostics-16-03236-s001.zip › diagnostics-4508874-supplementary.pdf]

Supplementary Table S1. Sex differences within each a priori age group

Age group	Sex	*n*	Mean ± SD	Median (IQR)
6–36 months	Male	32	2.98 ± 1.16	2.75 (2.10–3.47)
	Female	14	3.09 ± 0.99	3.20 (2.42–3.98)
37–72 months	Male	19	2.91 ± 1.27	2.90 (1.90–3.85)
	Female	24	3.56 ± 1.30	3.60 (2.49–4.33)
73–144 months	Male	33	3.97 ± 1.64	3.40 (2.80–5.20)
	Female	29	3.82 ± 1.40	3.30 (2.70–5.00)
≥145 months	Male	23	4.19 ± 1.89	3.40 (2.60–5.65)
	Female	14	3.59 ± 1.14	3.41 (2.62–4.52)
